# SCREEN: A simple layperson administered screening algorithm in low resource international settings significantly reduces waiting time for critically ill children in primary healthcare clinics

**DOI:** 10.1371/journal.pone.0183520

**Published:** 2017-08-29

**Authors:** Bhakti Hansoti, Alexander Jenson, Antony G. Kironji, Joanne Katz, Scott Levin, Richard Rothman, Gabor D. Kelen, Lee A. Wallis

**Affiliations:** 1 Department of Emergency Medicine, Johns Hopkins University School of Medicine, Baltimore, United States of America; 2 Department of International Health, Johns Hopkins University Bloomberg School of Public Health, Baltimore, United States of America; 3 Division of Emergency Medicine, University of Cape Town, Cape Town, South Africa; University of Miami, UNITED STATES

## Abstract

**Background:**

In low resource settings, an inadequate number of trained healthcare workers and high volumes of children presenting to Primary Healthcare Centers (PHC) result in prolonged waiting times and significant delays in identifying and evaluating critically ill children. The Sick Children Require Emergency Evaluation Now (SCREEN) program, a simple six-question screening algorithm administered by lay healthcare workers, was developed in 2014 to rapidly identify critically ill children and to expedite their care at the point of entry into a clinic. We sought to determine the impact of SCREEN on waiting times for critically ill children post real world implementation in Cape Town, South Africa.

**Methods and findings:**

This is a prospective, observational implementation-effectiveness hybrid study that sought to determine: (1) the *impact* of SCREEN implementation on waiting times as a primary outcome measure, and (2) the effectiveness of the SCREEN tool in *accurately* identifying critically ill children when utilised by the QM and *adherence* by the QM to the SCREEN algorithm as secondary outcome measures. The study was conducted in two phases, Phase I control (pre-SCREEN implementation- three months in 2014) and Phase II (post-SCREEN implementation—two distinct three month periods in 2016). In Phase I, 1600 (92.38%) of 1732 children presenting to 4 clinics, had sufficient data for analysis and comprised the control sample. In Phase II, all 3383 of the children presenting to the 26 clinics during the sampling time frame had sufficient data for analysis. The proportion of critically ill children who saw a professional nurse within 10 minutes increased tenfold from 6.4% to 64% (Phase I to Phase II) with the median time to seeing a professional nurse reduced from 100.3 minutes to 4.9 minutes, (p < .001, respectively). Overall layperson screening compared to Integrated Management of Childhood Illnesses (IMCI) designation by a nurse had a sensitivity of 94.2% and a specificity of 88.1%, despite large variance in adherence to the SCREEN algorithm across clinics.

**Conclusions:**

The SCREEN program when implemented in a real-world setting can significantly reduce waiting times for critically ill children in PHCs, however further work is required to improve the implementation of this innovative program.

## Introduction

In low resource international settings, it has been shown that decreasing delays in the identification and treatment of critically ill children can reduce mortality by up to 40%.[1] The top three causes of death in children under five years of age in Low and Middle Income Countries (LMIC), are pneumonia, diarrhea and newborn illnesses, which align with the top three complaints for children visiting primary healthcare clinics (PHC) in these same regions.[2] Most of these deaths (70%) can be prevented with simple and affordable interventions.[3] Due to an inadequate number of trained Health Care Workers (HCW) and high volumes of children presenting to PHC, waiting times remain high and often result in significant delays for critically ill children.

The WHO and UNICEF together created the Integrated Management of Childhood Illnesses (IMCI) strategy to improve the performance of trained HCW in PHC. IMCI is a clinical case management guideline that utilizes a syndromic approach to designate severity of illness and provide treatment recommendations. IMCI has been implemented in over 100 countries since its inception in 1996.[4] IMCI aims to guide care across a spectrum of childhood illnesses from children that are critically ill and warrant emergent transfer to those that present for preventative management and non-urgent complaints. The case management process requires a trained HCW (professional nurse equivalent) and between 5–7 minutes to complete per patient. In Cape Town, the clinics see between 100–150 children per day and thus it would take several hours for a child to see a professional nuse.[5] Given these inherent delays imposed by the IMCI guidelines, a pre-IMCI screening process became necessary to identify critically ill children at the point of entry into a clinic.

**Fig 1:**
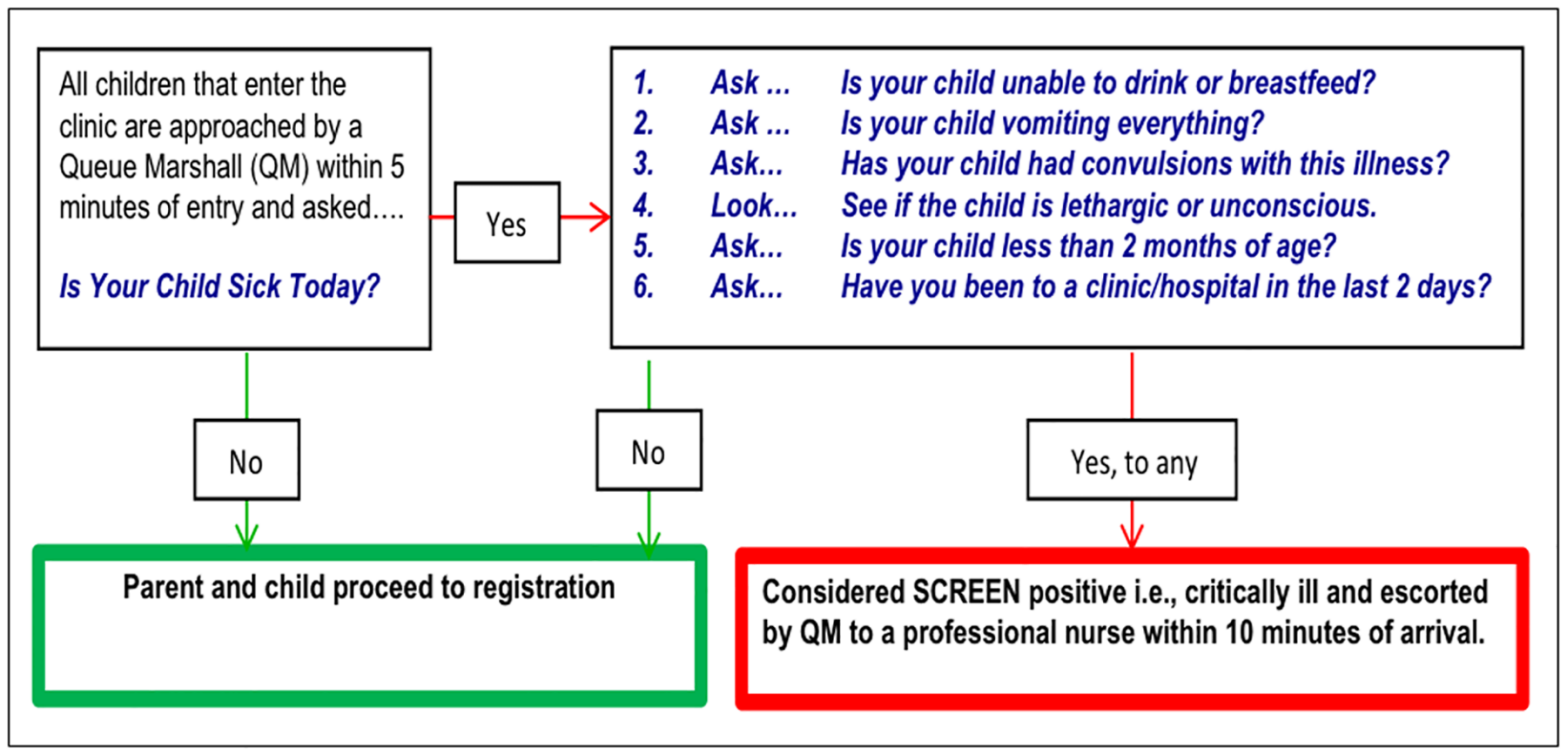
Infographic demonstrating the Sick Children Require Emergency Evaluation Now (SCREEN) program.

The Sick Children Require Emergency Evaluation Now (SCREEN) program was developed by the lead author (BH) in 2014, in collaboration with the University of Cape Town division of Emergency Medicine and the City of Cape Town Health Department. The purpose of SCREEN is to rapidly identify critically ill children and to expedite their care.[5,6] The program uses a six-question screening algorithm derived from the validated WHO IMCI danger signs for all children who report that they are “sick” on arrival (Fig 1).[7] Given the already overwhelming burden of work for HCW in the clinic setting, SCREEN was designed to be administered by Queue Marshals (QM), non-medical support staff recruited from the surrounding community providing administrative support to the clinic.[8] The SCREEN program requires the QM to question all children within 5 minutes of entry to the clinic and, if identified as SCREEN positive, to be taken to a nurse within 10 minutes of arrival to the clinic. Given the paucity of trained healthcare professionals in low resource settings, task shifting to QM may prove not only to be cost-effective but also more feasible to implement. Pilot studies have shown SCREEN to be highly effective in identifying and reducing the waiting times for critically ill children in PHC.[5,6] This study sought to determine the impact of SCREEN on waiting times for critically ill children post real world implementation in several PHCs in Cape Town, South Africa.

## Methodology

**Fig 2:**
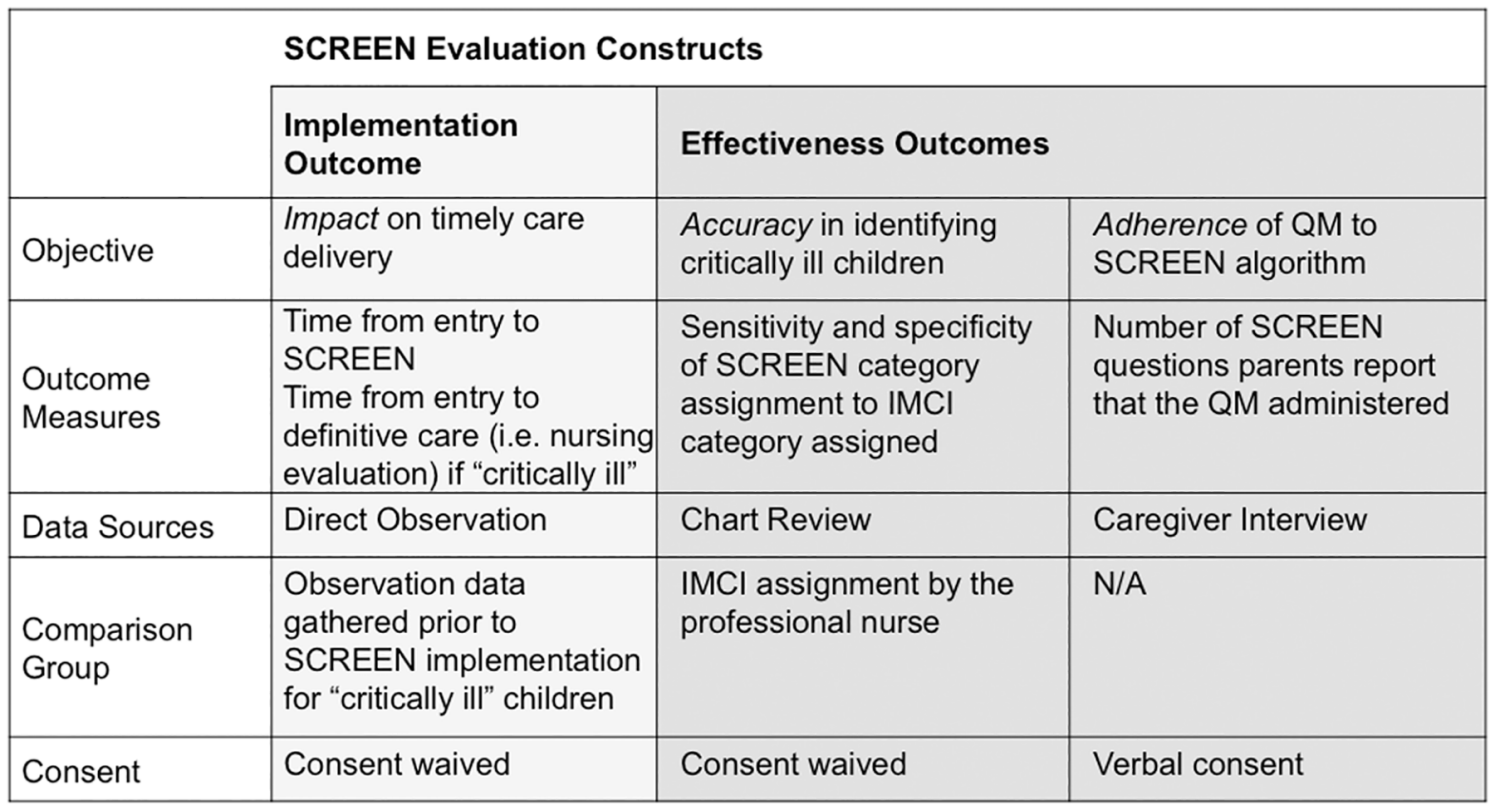
Overview of SCREEN implementation-effectiveness evaluation methodology.

This study is a prospective, observational implementation-effectiveness hybrid study that sought to determine: (1) the *impact* of SCREEN implementation on waiting times as a primary outcome measure, and (2) the effectiveness of the SCREEN tool in *accurately* identifying critically ill children when utilised by the QM and *adherence* by the QM to the SCREEN algorithm as secondary outcome measures.[9] Three distinct methodologies were utilized in the study design, the *impact* of the SCREEN program was evaluated using a direct observation methodology, the *accuracy* of the SCREEN tool was evaluated using a retrospective chart review methodology and *adherence* of the QM to the SCREEN algorithm was evaluated using caregiver interviews (Fig 2). The evaluation was conducted in two distinct phases, Phase I (pre-SCREEN implementation) and Phase II (post-SCREEN implementation).

### Setting

The study was conducted in select PHC caring for pediatric patients in Cape Town, South Africa. These clinics are open Monday to Friday for eight hours a day, and run by Nurses and clerks, who manage care without physician oversight. Professional nurses are tasked with the application of IMCI guidelines as part of their evaluation, and to identify critically ill children (designated as an IMCI “red” category) that need emergent transfer to a hospital for definitive care (often by ambulance). In many of the larger clinics a nursing assistant often sees a patient prior to the professional nurse, and is responsible for collecting vital signs and determining which queue the patients should wait in.[5]

### SCREEN implementation

The SCREEN program was widely implemented in PHC across the City of Cape Town in 2014. Training for QM was provided by the City of Cape Town IMCI training center. Given the overlap of the SCREEN tool with IMCI (Fig 1), a one day training program was created by adapting the IMCI training manual and included lectures, small group activities and role-playing.7 Training is provided twice year—in January and June—given the limited contracts for the QM. For the SCREEN program to function, each PHC was required to provide eight hours of coverage by a QM, who is tasked with standing at the entrance to each PHC, and surveying every child that enters using the simple SCREEN algorithm of yes or no questions (Fig 1). If any of the six SCREEN questions were positive, the QM was to immediately bring that child to be seen by a professional nurse. The nurse was to conduct a complete IMCI evaluation, administer any necessary treatment and facilitate transfer to a higher level of care if deemed necessary.

The QM salary is provided by the Expanded Public Works Program in South Africa, for a limited contract of six-months, which at the time of this study was compensated at ZAR 100 per day or approximately $1,900 per year for each clinic.[10,11]

### Outcomes measures

Primary Outcome Measure: The *impact* of SCREEN implementation on, “wait time” for “critically ill” children, defined as the time from clinic entry to time to see a professional nurse (Table 1). During Phase I, all IMCI designated “Red” children were considered “critically ill” as per standard practice at the time. During Phase II, all children designated by the QM as SCREEN positive (to any of six questions) were considered “critically ill”, as the standard of care in the clinic had changed post SCREEN implementation. Patients designated as IMCI “Yellow” or “Green” in Phase I, and SCREEN negative in Phase II were considered “not critically ill”. The goals of care in the clinics during both phases of the study were that all critically ill children must see a professional nurse within 10 minutes of arrival to a clinic. A goal specific to Phase II was that a QM evaluates each child presenting to a clinic within 5 minutes of arrival.

**Table 1:** Summary of patients enrolled by study phase and outcome measures.

		Phase I (Pre-SCREEN Implementation)		Phase II (Post-SCREEN Implementation)	
Data Collection Time Period		March 1^st^ to May 1^st^ (2014), N (%)		January 1^st^ to Mar. 31^st^, September 1^st^ to November 31^st^ (2016), N (%)	
**Primary Outcome Measures**	Number of Clinics/QM groups	4	-	26	-
	Total number of children who attended the clinic	1732	-	3383	-
	Total number children observed	1600	92.4%	3383	100.0%
	Total number of critically ill children^§^ (time analysis)	33	2.1%	170	5.0%
**Secondary Outcome Measures**	Accuracy: Total no of charts reviewed¶	n/a	-	827	24.4%
	Adherence: Total no of parents interviewed	n/a	-	977	28.9%
	Adherence: Total no of parents included in analysis*	n/a	-	493	50.5%

^§^ In Phase I critically ill is defined as IMCI RED, in Phase II critically ill is defined as any child who is determined as SCREEN positive based on the QM assessment (see text).

^**¶**^ Only charts that had both SCREEN documentation and IMCI documentation could be included in the chart review.

* Only parents who presented with a “sick” child who is not critically should have been asked all six questions and thus are included in the analysis.

Secondary Outcome Measures: The clinical effectiveness of SCREEN implementation was assessed in Phase II by two secondary outcome measures. First, *accuracy* of QM SCREEN designation was assessed by determining the sensitivity and specificity of QM SCREEN assignment (“positive” or “negative”) compared to the professional nurse assessment, using IMCI designation (“Red”, “Yellow” or “Green”) as the gold standard. Second, the *adherence* to implementing the SCREEN algorithm as intended was assessed by interviewing the accompanying caregiver with each child after the QM interaction. Each caregiver was approached soon after the SCREEN process. Following verbal consent to be interviewed, caregivers were asked to identify which, if any, of the six SCREEN questions they had been asked by the QM. Data was recorded on an electronic tablet using Magpi software (DataDyne, Washington, D.C.). SCREEN adherence by the QM was defined as the number of questions (out of a possible six) that each caregiver reported being asked by the QM. Only interviews from caregivers, whose children were reported as not “critically ill”, and thus eligible to be asked all six questions, were included in the analysis (Table 1).

### Participants

The study was conducted in two phases (Table 1). Phase I (pre-SCREEN implementation) participants were from a subset of four PHCs and served as historic controls.[5] Phase II (post-SCREEN implementation), participants were from a subset of 13 PHCs chosen by the City of Cape Town executive health management team, participating clinics had employed a QM and implemented the SCREEN program. At the time of this study, the SCREEN program had only undergone pilot implementation, and thus only a handful of the 208 PHCs within the city of Cape Town had been funded, by the city of Cape Town executive health management team to participate, special care was taken to ensure geographic and socio-economic diversity across the city.

### Data collection

Primary Outcome Measure (*Impact* of SCREEN on wait time): Phase I control data were collected from March 1st to May 1st 2014. Each of the four clinics was sampled for five consecutive weekdays. The clinics were chosen using a convenience sample.[5] Control data were collected via a custom developed Android based smart-phone application designed for use by clinic staff to track patient flow in the clinics. Each child who presented to the clinic was allocated a randomly generated four-digit number, encoded in a quick response (QR) code sticker and placed on the child's clothing prior to entering the clinic. Every time a staff member would scan the QR code a ‘time of scanning’ and the four-digit number were uploaded to a cloud-based database. Staff routinely scanned the QR code at (1) time of entry into the clinic, (2) time seen by any healthcare staff member, (3) time seen by a professional nurse and (4) time of exit from the clinic. The application also allowed the nurses to denote the severity of illness of the child using the IMCI designation. For the purpose of data analysis, we utilized (1) the time the child entered the clinic and (3) the time the child saw the professional nurse. This methodology is presented in greater detail in our pilot study.[5]

Phase II data were collected from each of the 26 clinics from Jan 1^st^ to March 31^st^ 2016, and from September 1^st^ to November 31^st^ 2016. The study was conducted in two different time periods to capture clinics in both the winter and summer months in South Africa. Each clinic was sampled for two consecutive weekdays within the two-time frames. Our goal for the post implementation study was to maximize the number of clinics included. Direct observation of the QM-patient interaction at the entry point into the clinic, was conducted by investigators (BH, AJ, AK) and supervised local research staff trained by BH. This methodology was used to capture (1) time of entry in to the clinic, (2) time of screening by QM, and (3) time of professional nurse evaluation if SCREENed as “critically ill”. Each clinic was assigned two or three observers and thus direct observation was maintained throughout the day. Data were recorded using Microsoft Excel software on an electronic tablet. The QM and nurses were blind to the purpose of the observation, and efforts were taken to physically distance the observer from the QM. Data collection was identity unlinked i.e., no data on age, sex and chief compliant were collected, and the observer did not approach the patients/parents during the interaction.

Secondary Outcome Measures (*Accuracy* of the SCREEN tool by QM and *adherence* to the SCREEN tool): In Phase II *accuracy* of the SCREEN program was evaluated using a retrospective chart review methodology. Only charts that had both the SCREEN designation and IMCI designation were included. Chart reviews were conducted on the same day as the assessments. *Adherence* of the QM to the SCREEN algorithm was evaluated using caregiver interviews. Attempts were made to interview as many caregivers as possible soon after the SCREEN intervention to determine how many of the questions they were asked by the QM. Interviews were conducted by assistants not involved in the timing events. In Phase II it was not possible to connect the three data sources (observation, chart-review and interview). However, all data were collected during the same sample time frame.

### Sample size calculations and data analysis

Primary Outcome Measure (*Impact* of SCREEN on wait time): The sample size calculation for Phase II of the study is based on the pilot data gathered in Phase I. During Phase I, the median “wait time” to see a professional nurse was 100.3 minutes. The SCREEN program is designed to reduce the “wait time” for all critically ill children to less than 10 minutes. Given this effect size (0.9), we would need to recruit a total of 39 critically ill children, to obtain an alpha of .05 and power of 80%. Thus, for Phase II we calculated a need to observe 1858 children to capture 39 “critically ill” children. Using STATA v.12 (StataCorp, LLC, Texas), a cox-regression was constructed for the time-series of “wait time” to see a professional nurse for “critically ill” children (IMCI “Red” children in Phase I and SCREEN “positive” children in Phase II) with SCREEN implementation as the covariate. The proportion of SCREEN positive children who saw a nurse within 10 minutes was also calculated. ANOVA testing was used to detect inter-clinic variability in time to screening and time to seeing a professional nurse.

Secondary Outcome Measures (*Accuracy* of the SCREEN tool): Based on WHO burden of disease data^12^ and pilot data^6^ we assumed that the prevalence of critically ill children would be between 5–10%. The sensitivity, specificity, and predictive values (negative and positive) of SCREEN were calculated by cross tabulating QM SCREEN designation to nurse designated IMCI category, which was made binary, i.e., either “Critically Ill” (IMCI Red), or “Not Critically Ill” (IMCI Green or Yellow). For the accuracy analysis, we determined a sample size of 118 critically ill children will be required for the chart review to have a power of 80%, to detect a sensitivity of 95% or higher, assuming a significance level 0.05 (one-sided), and power of 80%.

### Ethical considerations

Both the Johns Hopkins University School of Medicine Institutional Review Boards and the University of Cape Town Health Research Ethics Committee approved the study. In addition, we received approval from the City of Cape Town Health Department.

## Results

**Fig 3:**
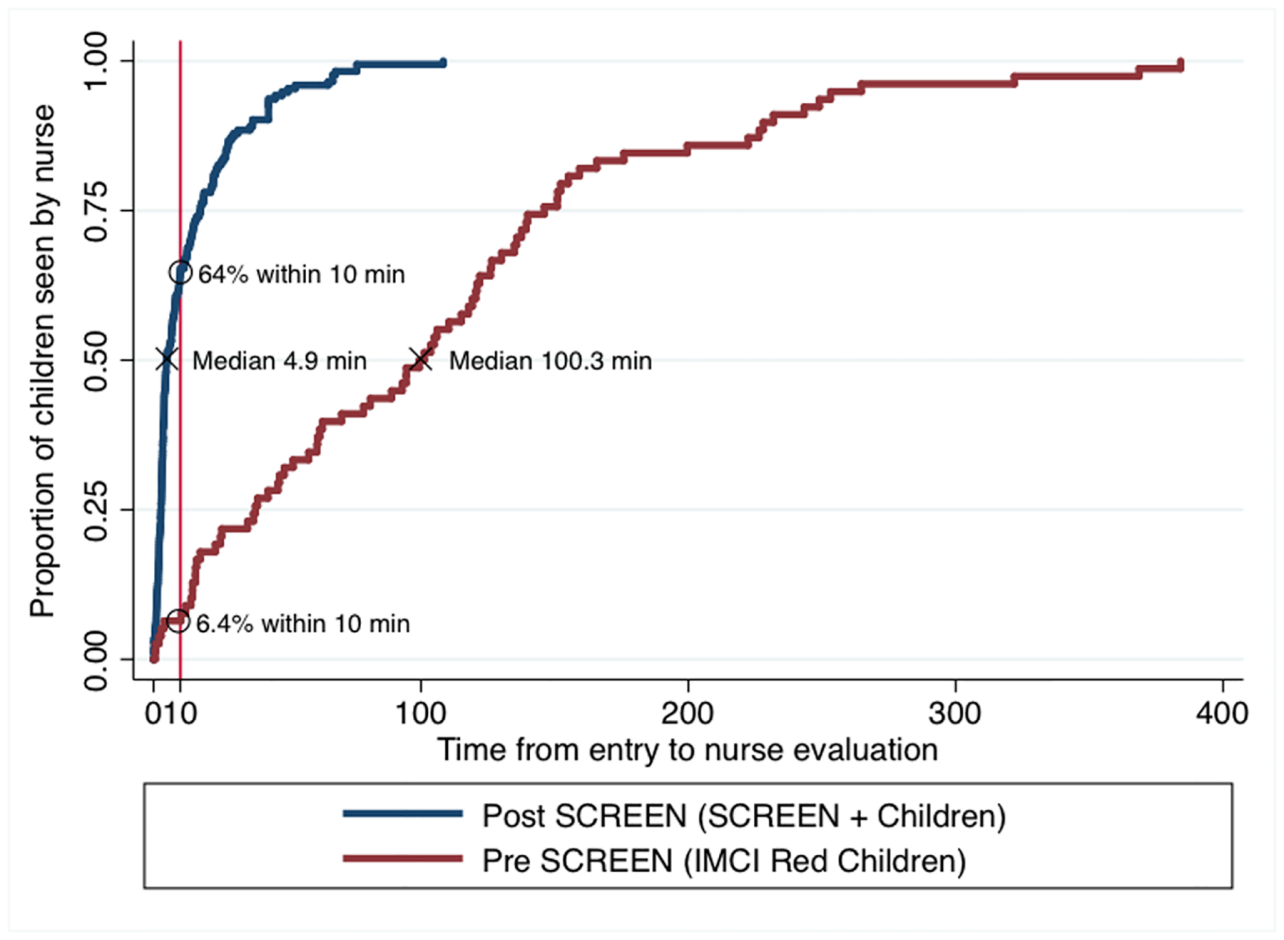
Pre-and-post impact of SCREEN implementation on wait times to nurse evaluation for critically ill children, Kaplan Meier survival analysis.

For the *impact evaluation*, a total of 1732 children were enrolled in Phase I and 3383 children were enrolled in Phase 2. In Phase I, of the 1732 children presenting to the 4 clinics, 1600 (92.38%) had sufficient data for analysis and comprised the control sample. In Phase II, all 3383 of the children presenting to the 26 clinics during the sampling time frame had sufficient data for analysis (Table 1). During Phase I, 33 (2.1%) children were designated as IMCI “Red” while in the Phase II, 170 children (5.1%) were designated as SCREEN positive (Table 1). The proportion of critically ill children who saw a professional nurse within 10 minutes increased tenfold from 6.4% to 64% (Phase I to Phase II) and the median time to seeing a professional nurse reduced from 100.3 minutes to 4.9 minutes, respectively (Fig 3).

There was however a large variance in the proportion of children that were able to meet the SCREEN program goals of care by clinic. In Phase II only 3,049 (90.1%), of the 3383 children presenting for care, were evaluated by a QM. The average proportion screened within five minutes varied greatly across clinics (median 84.1%, IQR 66.3%-90.3%, ANOVA R = 0.2859, p<0.0001) and the average proportion of SCREEN positive children seeing a professional nurse within ten minutes also varied across clinics (median 83.5%, IQR 38.8%-100%, ANOVA R = 0.3936, p<0.0001).

*Accuracy* of the SCREEN tool was measured by retrospective chart review. A total of 827 (24.4%) patient charts had both SCREEN and IMCI designations and were thus audited for accuracy (Table 2). Overall, QM screening compared IMCI designation had a sensitivity of 94.2% and a specificity of 88.1% (Table 3). The SCREEN tool when implemented by the QM had a positive predictive value of 26.0% and a negative predictive value of 99.7% (Table 3).

**Table 2:** Characteristics of children included in the retrospective chart review.

	Jan-March 2016	Sept-Nov 2016	Total/Overall
Clinics/QM groups	13	13	26
Total (N)	486	341	827
Months of Study	Jan-March (Winter)	Sept-Nov (Summer)	6 months
Age (mean months)	30.2	23.1	27.6
Male Sex—N (%)	250 (51.6%	154 (45.2%)	404 (48.9%)
Temp (mean degrees Celsius)	36.5°	36.5°	36.5°
Fever > 38 degrees Celsius—N (%)	27 (5.6%)	41 (12.0%)	68 (8.2%)
Below Weight < 25^th^ percentile- N (%)	28 (5.8%)	11 (3.1%)	39 (4.7%)
IMCI Green	290 (59.7%)	255 (74.8%)	545 (65.9%)
IMCI Yellow	169 (34.8%)	78 (22.8%)	247 (29.9%)
**IMCI Red**	27 (5.6%)	8 (2.3%)	35 (4.2%)
**SCREEN positive n (%)**	76 (15.6%)	51 (15.0%)	127 (15.4%)
**IMCI RED missed by SCREEN**	0	2	2 (0.0%)

**Table 3:** Sensitivity and specificity analysis.

		IMCI*		
		Red	Yellow/Green	N
**SCREEN**	Positive	33	94	127
	Negative	2	698	700
	N	35	792	827

*IMCI = Integrated Management of Childhood Illnesses, IMCI-Red = Critically Ill, IMCI-Yellow/Green = Not Critically Ill.

A total of 977 caregivers were approached for interview post SCREEN to measure *adherence* of the QM to the SCREEN algorithm. All caregivers consented to be interviewed, of these, 493 (50.5%) reported presenting with a “sick” child that was not critically ill and thus should have been asked all six questions. Of these, only 23% of caregivers answered “yes” to being asked all six of the SCREEN questions. There was high variability in the average number of questions asked per clinic (median 3.4, IQR 2.9–4.2, ANOVA R = 0.2452, p<0.0001). The least asked question (44% of encounters) was “Has your child being seen in a clinic/hospital in the last 2 days?” while the most commonly asked question (77%) was, “Is your child vomiting everything?”. The raw data utilized for all the analysis presented in the results section is availiable in a supporting information file (S1 Dataset).

## Discussion

Interventions can face major obstacles when scaled up.[12,13] Despite a strong technical basis for the IMCI program, multi-country evaluations have revealed mixed results regarding the success and implementation of IMCI.[14] Problems with competing commitments, coordinated program management, and supervision impact the delivery of healthcare interventions.[15,16,17] Understanding the delivery gaps in implementing the SCREEN program in a real-world environment will inform the further dissemination of this innovative program. Overall our study found that the SCREEN program performed well in a real-world environment and was able to significantly reduced the wait time for critically ill children to be definitively evaluated by a professional nurse.

Our study suggests, that a simple screening tool for use by laypersons, can be successfully implemented in a primary healthcare setting to prioritize the care of critically ill children in low resources environments. We did however identify that there were large variances in meeting the SCREEN program goals across sites. While overall the waiting time analysis is extremely promising, in some clinics, none of the critically ill children met the goal of being expedited to a professional nurse within ten minutes. This may be due competing duties placed on the QM or a lack of ongoing supervision and review of QM performance.

The most important criteria for a screening tool is sensitivity. Using IMCI as the gold standard, the SCREEN tool, as administered by lay staff, proved highly accurate (high sensitivity and specificity) in identifying critically ill children. The tool did have a low positive predictive value (PPV) resulting in many children being expedited that were not in-fact critically ill. The PPV is likely a result of the low prevalence of critical illness in this setting. The proportion of children identified, as critically in our study was 5%, which is significantly higher than what is reported in the high-income country literature.[18–20] Despite the low frequency of serious infection, the need to develop a strategy that provides early identification is universally accepted.[21–24] Most work in resource-limited settings has focused on the use of trained HCWs.[23] However, given the paucity of trained healthcare providers, and relative low volume of critically ill children, a simple tool that can be implemented by lay providers offers a feasible, financially responsible, solution.

Despite training and the linguistic similarities of the SCREEN tool to the IMCI danger signs we found a large variance in the completeness of use of the SCREEN algorithm. Other studies that look at the adherence of healthcare workers to IMCI danger signs also show similarly low adherence, with only 6.6% of healthcare workers asking parents about three or more of these crucial danger signs. [25] In an observational study in Benin on average only 1 out of the 4 danger signs were assessed per child. [26] It is unclear what factors drove the low adherence of the QM to the SCREEN algorithm. We hypothesize that adherence to the SCREEN questions may be poor, as perhaps the QM intuitively/ instinctively only asked certain questions, i.e., that some QM seeing an interactive/well appearing child in the clinics did not feel the need to ask the questions prescribed as they felt they did not apply. Overall the evaluation for adherence was disappointing. Despite poor adherence to the actual questions, the SCREEN tool remained sensitive in identifying sick children. Similar to our study IMCI has also been proven to have a large impact despite variable adherence in practice. The success of the SCREEN program is perhaps secondary to the interaction with the QM (looking at the child and speaking to the caregiver) which may have a more significant impact on identifying a critically ill child than the questions themselves. Regardless, we anticipate that adherence may be significantly improved with reinforced training and supervision, which may improve the specificity over time. [26–28]

The success of the SCREEN program in Cape Town speaks to the strong research and local stakeholder relationship that was built during the course of this research. Over the two-year period from the start of the project (that resulted in the SCREEN program development) and the conclusion of this study, the study team met with the City of Cape Town executive health management team regularly to provide real time access to the SCREEN data and discuss strategies for future implementation. This real-time access to the pilot studies likely had the biggest impact on the successful implementation of the SCREEN program as it allowed policy makers to see the gaps in the current healthcare system and the benefit of this successful intervention. This led to the SCREEN program proceeding from development to full implementation in less than two years. Since fully implemented the SCREEN program has been adopted by the City of Cape Town executive health management team who have decided to fund this important program in clinics with high volumes. Training responsibilities are assigned to the City of Cape Town IMCI training center, given the similarities of SCREEN to IMCI. To date, SCREEN remains an integral component of care delivery across PHC in Cape Town, South Africa.

## Limitations

This study was conducted in real world conditions where it is hard to control for many factors. This is a post implementation research study in a complex clinic based system where observations and data collection were designed to not interfere in processes or disturb care. To allow minimal impact we chose not to collect data on the children themselves.

Due to our sampling strategy nine clinics in phase II were sampled in both the summer and winter. Our choice of clinics was restricted to those that had committed to funding the SCREEN program in this early phase. The QMs employed in each of the 26 evaluations however are unique. Our analysis revealed no difference in the performance of QMs from clinics that were sampled twice opposed to just once, thus the data in this study is presented as aggregate.

During the study, it is likely that the QM, nurses and other clinic staff were aware that the research team was making observations. Given the relatively short period of observation, the Hawthorne Effect may have skewed results toward a favorable outcome. Given the variability of results across the PHC sites, this effect, if present, certainly wasn’t uniform.

For our secondary outcome measures, only about 25% of the children who presented for care were included in the accuracy study. Although we did not keep track, a sizable proportion of children present for care to the clinic for well-child checks and routine immunizations. In these cases, it was not necessary for the nurse to complete IMCI documentation. It is also possible that some charts may have been missed due to current use, or review by pharmacy. Similarly, we may have been unable to interview all caregivers, given that many clinics have numerous waiting rooms, and parents come and go during the day. Finally, the interviews relied on the recall of caregivers, while every attempt was made to speak to the caregiver immediately after their interaction with the QM we anticipate some recall bias.

## Conclusions

The SCREEN program effectively reduces waiting times for critically ill children in the primary healthcare setting. Despite poor adherence to the full SCREEN algorithm, having a QM at the point of entry to the PHC produces accurate and timely expedition of care for critically ill children. Future work needs to focus on developing a population-based study that can evaluate the cost effectiveness and long-term sustainability of such an intervention. To understand the true impact of the SCREEN program we need to evaluate if such a program can be successfully scaled up and if that program has an impact on overall childhood mortality in resource limited environments.

## Supporting information

S1 Dataset Plos one SCREEN de-identified raw data. (XLSX)
